# Factors related to poor adherence in Latvian asthma patients

**DOI:** 10.1186/s13223-020-0414-6

**Published:** 2020-03-04

**Authors:** Dins Smits, Girts Brigis, Jana Pavare, Inga Urtane, Sandis Kovalovs, Noël Christopher Barengo

**Affiliations:** 1 Faculty of Public Health and Social Welfare, Riga Stradins University, Marupes 30, Riga, 1002 Latvia; 2 Faculty of Medicine, Riga Stradins University, Riga, Latvia; 3 Faculty of Pharmacy, Riga Stradins University, Riga, Latvia; 4 Department of Translational Medicine, Division of Medical and Population Health Sciences Research, Herbert Wertheim College of Medicine, Florida International University, Miami, FL USA; 5 Department of Public Health, Faculty of Medicine, University of Helsinki, Helsinki, Finland

**Keywords:** Medication adherence, Cognition, Outpatients, Asthma, Latvia

## Abstract

**Background:**

The problem of nonadherence to therapy is a key reason of insufficient asthma control. Evaluating the beliefs about asthma medication, cognitive and emotional perceptions may help to identify patients with poor adherence to treatment in clinical practice which need additional attention in order to increase the likelihood of them taking their asthma medication according to the prescribed treatment protocol. The purpose of this study is to assess whether beliefs about asthma medication, cognitive and emotional factors are related to poor treatment adherence of asthma medication in a sample of asthma patients in Latvia.

**Methods:**

Study subjects were asthma patients attending outpatient pulmonologist consultations in Latvia during September 2013 to December 2015. Beliefs about asthma medicine, cognitive and emotional factors related to asthma were determined in a cross-sectional, self-administered survey. The validated Beliefs about Medicines Questionnaire (BMQ) and the Brief Illness Perception Questionnaire (brief IPQ) were used. Treatment adherence was assessed using 5-item version of the Medication Adherence Reporting Scale (MARS). The total sample size was 352 patients. Logistic regression models were used to predict poor adherence to asthma treatment. The validity of each logistic regression model was assessed by the Hosmer/Lemeshow test. The main outcome measure was self-reported adherence to treatment.

**Results:**

The more the patients agreed with the statement “My future health depends on my asthma medication” the lower the possibility of poor adherence to asthma treatment (OR 0.42; 95% CI 0.24–0.74). The more concerned the patients were in regard to long-term effects of their medication (OR 2; 95% CI 1.22–3.27), the higher the probability of poor treatment adherence.

**Conclusions:**

Screening asthma patients using the BMQ may help to identify those to benefit from interventions targeting their concerns and medication beliefs in order to improve adherence to asthma medication.

## Background

The problem of non-adherence to the therapy is one of the main reasons of insufficient asthma control [1–3]. Studies assessing treatment plans in asthma patients have revealed that adherence to medication range between 30 and 70% [4–6]. Several factors related to the patient, to the disease, treatment or physician–patient relationship have been identified to be related with adherence in asthma and other diseases [7–12]. According to the self-regulation theory, both illness perception and medication beliefs are associated with medication adherence [13, 14]. Particularly beliefs about illness, the necessity and the concerns (side effects and addition) of the pharmaceutical treatment have been identified to be the two most important elements in the proposed theory [13–16]. Other factors that impact adherence to treatment are cost of therapy, perceived efficacy of medicines, complex dosing regimens. Cost of treatment might be of particular concern for asthma patients in Latvia since the reimbursement level for asthma medication is 75% of the total medication cost.

Evaluating the beliefs about asthma medication, cognitive and emotional perceptions may help to identify patients with poor adherence to treatment in clinical practice. That would guide additional attention helping to increase the likelihood of taking asthma medication appropriately. However, only limited information is available on treatment adherence in Latvian asthma patients. Furthermore, evidence identifying medication beliefs, cognitive or emotional factors associated with asthma medication adherence among Latvian population is currently not available.

## Aim of the study

The aim of this study was to investigate whether beliefs about asthma medication, cognitive and emotional factors are related to poor adherence of asthma medication in a sample of Latvian asthma patients.

## Method

### Study population

The study population of this cross-sectional patient survey consisted of asthma patients attending outpatient pulmonologist consultations in Latvia. The data were gathered during a period of 28 months from September 2013 to December 2015. In a first step, a list of all pulmonologists from the database of the National Health Service of the medical doctors that have contractual rights to prescribe reimbursed medicines was acquired. Then, pulmonologist practices in medical centres and hospitals in Riga and in bigger towns of Latvia were selected and invited to join the study. 15 of these practices agreed to participate (convenience sample). These 15 medical centres employ 66 pulmonologists that is 51% of all 129 pulmonologists in Latvia that have a contract with NHS. We assumed these 15 practices to be representative of all pulmonologist practices in Latvia. Each pulmonologist was advised to invite their patients to join the survey. Patients’ age group were 15 years and older. Patients using controller medication–inhaled glucocorticoids in monotherapy or in combination with long acting beta 2 mimetic medicines for at least 12 months prior to the study were included. The sample size was calculated to detect a prevalence of poor asthma control of 50% with a margin of error of 5%, and a power of 95%. The total sample size needed was 352 people.

### Assessment of main variables

A self-administered questionnaire was used to assess socio-demographic and economic factors such as age, education, income and sex.

Adherence to asthma medication was assessed using 5-item version of the Medication Adherence Reporting Scale (MARS). MARS is a validated questionnaire that has shown to have good internal, construct and criterion validity, including correlations with objective measures of adherence (electronic monitoring and pharmacy dispensing data) [6, 17]. The short version of the MARS is a five-item self-report scale for assessment of adherent behavior that includes assessment of:

- unintentional non-adherent behavior
  “I forgot to take them”, (item 1) and
- intentional non-adherent behavior
  “I alter the dose”, (item 2).
  “I stop taking them for a while”, (item 3).
  “I decide to miss out a dose”, (item 4).
  “I take less than instructed”, (item 5).

Each item was answered using a five-graded response scale, ranging from very often (1) to never (5). Low scores indicate low levels of adherent behavior [18].

Medication beliefs were assessed using the five items of greatest relevance to asthma medication adapted from the BMQ, a validated tool across many disease conditions [19]. The specific–necessity scale contains 5 items that assess patients’ beliefs about specific necessity to take prescribed chronic medications. All three questions assessing patients’ beliefs about specific necessity to take prescribed chronic medications or concerns were selected from the original BMQ. All belief items had Likert scale responses.

The Brief Illness Perception Questionnaire (brief IPQ) was used to obtain information on illness perception of the study participants. The Brief IPQ consists of eight items and a causal question [20, 21]. All of the items except the causal question are rated using a 0-to-10 response scale. Five of the items assess cognitive illness representations: consequences (item 1), timeline (item 2), personal control (item 3), treatment control (item 4), and identity (item 5). Two of the items assess emotional representations: concern (item 6) and emotions (item 8). One item assesses illness comprehensibility (item 7). Assessment of the causal representation is by an open-ended response, which asks patients to list the three most important causal factors in their illness (item 9).

### Statistical analysis

The Statistical Package for the Social Sciences (SPSS) IBM 21.0 was used to analyse the data. Means, standard deviations, and frequencies are presented to describe the characteristics of the study sample. A cutoff point of > 6 for MARS-5 was used to define poor medical adherence.

The answers of the BMQ were dichotomized into (i) “I agree/I completely agree” and (ii) “Not sure/I disagree/I completely disagree”. The logistic regression analyses were first conducted for each variable alone. In the multivariate logistic analysis, the outcome variable was controlled for age, income and educational level. The odds ratio and respective 95% confidence interval are presented for all models. The validity of each logistic regression model was assessed by the Hosmer/Lemeshow test.

## Results

The baseline characteristics of the study population are presented in Table 1.

**Table 1:** Baseline characteristics of the study sample

	Men	Women	Total
	(n = 85)	(n = 264)	(n = 352)
Age, mean (SD^a^)	53.7 (17.4)	58.7 (16.6)	57.5 (16.9)
Education%			
Basic	3.5	7.5	6.5
Secondary	23.3	27.1	26.1
Vocational	47.7	28.9	33.5
Higher	25.6	36.5	33.8
Income%			
< 300 €/month	16.7	25.3	23.2
300–550 €/month	36.9	48.2	45.5
550–750 €/month	28.6	18.3	20.8
> 750 €/month	17.9	8.2	10.6
Asthma medication%			
Glucocorticoids	62.8	63.4	63.3
Glucocorticoids+beta 2 mimetic	33.7	33.2	33.3
Poor treatment adherence% (MARS-5 item scale)	58.1	68.7	66.1

^a^Standard deviation

The majority of the patients had vocational education and were earning at least 300 euros per month. Two out of three patients were using glucocorticoids and one-third a combination therapy consisting of glucocorticoids and a beta 2 mimetic drug. The prevalence of poor treatment adherence was 58.1% in men and 68.7% in women.

We observed that monotherapy with corticosteroids reduced the odds of poor treatment adherence whereas fixed-dose combination medications containing a corticosteroid and a beta 2 mimetic increased the odds of poor treatment adherence.

None of the socio-demographic or socio-economic factors were predictors of poor treatment adherence (Table 2). Vocational level of education seemed to increase the odds to have poor treatment adherence.

**Table 2:** Odds ratio of socio-demographic and economic factors in regard poor treatment adherence

	MARS 5-item scale	
	Univariate	
	OR^a^	(95% CI^b^)
Age	0.98	(0.97–1.00)
Female sex	1.58	(0.96–2.60)
Education		
Basic or secondary	1	ref
Vocational	1.79	(1.03–3.11)
Higher	1.27	(0.75–2.16)
Income		
< 300 €/month	1	ref
300–550 €/month	0.86	(0.48–1.55)
550–750 €/month	0.80	(0.41–1.59)
> 750 €/month	0.87	(0.38–2.03)
Asthma medication		
Corticosteroids	0.57	(0.35–0.91)
Corticosteriods+beta 2 mimetic	1.93	(1.18–3.16)

^a^Odds ratio

^b^Confidence interval

Table 3 shows the odds ratio of the association between cognitive and emotional illness indicators with poor treatment adherence. Apparently, none of these indicators were able to predict poor treatment adherence in Latvian asthma patients.

**Table 3:** Odds ratio of the association of cognitive and emotional illness indicators and poor treatment adherence

	MARS 5-item scale			
	Univariate		Mutivariate^c^	
	OR^a^	(95% CI^b^)	OR	(95% CI)
How much does your illness affect your life?	0.96	(0.88–1.05)	0.96	(0.88–1.05)
How long do you think your illness will continue?	0.96	(0.87–1.05)	0.98	(0.89–1.08)
How much control do you feel you have over your illness?	0.98	(0.89–1.08)	0.98	(0.88–1.08)
How much do you think your treatment can help your illness?	0.96	(0.86–1.07)	0.96	(0.85–1.07)
How much do you experience symptoms from your illness?	1.01	(0.93–1.10)	1.01	(0.92–1.11)
How concerned are you about your illness?	0.99	(0.93–1.07)	1.01	(0.94–1.09)
How well do you feel you understand your illness?	1.06	(0.98–1.15)	0.98	(0.97–1.15)
How much does your illness affect you emotionally?	1.03	(0.96–1.10)	1.03	(0.96–1.11)

^a^Odds ratio

^b^Confidence interval

^c^Adjusted for age, education and income

Several beliefs about medication were identified as predictors of poor treatment adherence (Table 4). Asthma patients who were convinced that their future health depended on the asthma treatment were less likely to have poor treatment adherence (OR 0.42: 95% CI 0.24–0.74). In case the patient was concerned by the long-term effects of their asthma medication the odds of poor treatment adherence were 2 (95% CI 1.22–3.27). Furthermore, patients who were concerned by the need to constantly use their asthma medication seemed to have increased odds to have poor treatment adherence, but the association was not statistically significant.

**Table 4:** Odds ratio of the association between medication beliefs and poor treatment adherence

	MARS 5-item scale			
	Univariate		Mutivariate^c^	
	OR^a^	(95% CI^b^)	OR	(95% CI)
Necessity				
My health is fully dependent on the asthma medication	0.58	(0.36–0.94)	0.61	(0.37–1.01)
Without asthma medication my life would be impossible	0.64	(0.41–1.01)	0.62	(0.38–1.01)
Without my asthma medication I would be very ill	0.71	(0.45–1.12)	0.72	(0.44–1.19)
My future health depends on my asthma medication	0.42	(0.25–0.70)	0.42	(0.24–0.74)
My controlling asthma medication prevents health deterioration	0.51	(0.28–0.96)	0.61	(0.32–1.17)
Concerns				
I am concerned by the need to constantly use my asthma medication	1.69	(1.07–2.68)	1.59	(0.98–2.57)
I am sometimes concerned by long term effects of my asthma medication	1.89	(1.19–3.01)	2	(1.22–3.27)
My asthma medication is incomprehensible to me	1.03	(0.60–1.79)	1.27	(0.70–2.29)

^a^Odds ratio

^b^Confidence interval

^c^Adjusted for age, education and income

In addition, we conducted a correlation analysis between the two variables of the BMQ questionnaire that were statistically significant predictor of treatment adherence. However, no statistically significant correlation was observed (r = 0.068; *p* value 0.199).

## Discussion

Our study revealed that several beliefs about asthma medication were predictors of poor treatment adherence in Latvian asthma patients. However, none of the indicators of illness perception seemed to be associated with suboptimal adherence. Moreover, it is of concern that only three out of ten asthma patients in Latvia had adequate treatment adherence during the timeframe of our study.

Latvia differs from other countries in access to medicines. The reimbursement level for asthma medication in Latvia is 75% of the pharmacy price and therefore patient’s co-payment for a monthly supply of a controller medication was 1452 EUR for the most widely used medicine at the time of the study. It is a significant amount relative to the reported income of our study subjects. We may speculate that significant financial burden is a factor of low total adherence in our study. There have been previous studies that look at this factor. A study in US assessed impact of a full reimbursement of medication used after myocardial infarction compared to a usual reimbursement plan [22]. The results showed 4 to 6 percentage points higher adherence rate in the full-coverage group.

The prevalence of poor adherence in Latvian asthma patients is higher than reported in previous research [6–8] whereas poor adherence to asthma treatment was found to be 60% in the USA and was ranging between 30 and 40% in France, Germany and Italy [6]. Several intentional and non-intentional factors have been shown to be related to poor treatment adherence [7–9]. Some factors are related to the patient, others to the disease, treatment or physician–patient relationship [10, 11].

Patients’ medication beliefs may explain a significant portion of variation in medication non-adherence in asthma patients [12]. Our results in regard beliefs about medication and treatment adherence are in line with previous findings showing that beliefs about the necessity and the concerns about side effects of the asthma treatment are well associated with poor adherence (Leventhal et al. 2005); [14–16]. A British study in 100 community-based patients showed that non-adherent behaviours were associated with doubts about the necessity of medication and concerns about its potential adverse effects [14]. Furthermore, a cross-sectional survey among 238 Dutch asthma patients also reported that self-reported adherence correlated significantly with necessity beliefs and concerns of chronic use of inhaled corticosteroids [16]. These beliefs and concerns against perceptions of necessity have been found to predict adherence to medication not only in asthma patients but also in treatment of HIV, arthritis and coronary heart disease [15, 16, 23, 24]. In addition, current evidence suggests that certain beliefs about medicines are more predictive of intentional non-adherence than of unintentional non-adherence [15]. For instance, in a study among asthma patients in New Zealand intentional non-adherence (missing or altering doses to suit one’s needs) is associated with decision balance, whereas unintentional non-adherence (forgetting to take medication) is less strongly associated with decision balance and is more strongly associated with demographics, in particular age [18].

Patients often make treatment choices according to their own understanding and beliefs about the illness and treatment. Patients’ adherence to medication is particularly influenced by the way in which they evaluate their personal need for medication relative to their concerns about potential negative effects of taking it. Our finding that using fixed-dose combination medications containing a corticosteroid and a beta 2 mimetic increased the odds of poor treatment adherence might seem a paradoxical result since fixed dose combination medicines of inhaled corticosteroid and bet 2 mimetic offer treatment effect that a patient can feel, whereas effects of plain corticosteroid a patient would not usually feel as it will reduce number of disease exacerbations over time. However, we may speculate that the symptom relieving effect of the beta 2 mimetic component of the medicine is indeed causing non-adherent behaviour. Patients would feel their symptom relief brought by the beta 2 mimetic component of their medicine and that would shift the necessity-concern balance in the patient’s perception. They would decide that their disease is not that severe anymore and the resulting action would be taking less medicine.

National Institute of Clinical Excellence (NICE) guidance recommends addressing any beliefs and concerns that patients have that result in reduced adherence by uncovering individual’s perceptions of their medication during the consultation [25]. BMQ is a useful tool to assess medication beliefs and concerns and we recommend using at least some questions of BMQ routinely if not all of the questionnaire in the clinical practice.

The self-regulatory model was developed in order to explain illness-related behaviour, including adherence to treatment recommendations within the field of non-communicable diseases [13]. According to that model adherence to treatment consists of several components that the patient can adopt to cope with their disease. Only a few studies have assessed the association between illness perception and treatment adherence in asthma patients [14, 26]. In contrast to our study, non-adherent behaviours were associated with more negative perceived consequences of illness in a British study conducted among 100 asthma patients [14]. Furthermore, in a large Polish survey of 3618 asthma patients, illness perception, younger age, disease duration and severity were predictors of adherence to treatment with fluticasone propionate and formoterol fumarate [26]. It has also been suggested that interventions focusing on illness perception helped to support chronic obstructive pulmonary disease patients in their disease management and to improve health-related quality of life [27]. More evidence is needed to show whether the same principle applies for asthma patients.

Naturally, several limitations may be considered. Asthma medication adherence was measured by a self-report questionnaire not previously validated in the Latvian population. Self-reported measurements of medication adherence may not be that precise and are subject to self-presentational and recall biases. However, MARS is a validated questionnaire that has shown to have good internal, construct, and criterion validity, including correlations with objective measures of adherence (electronic monitoring and pharmacy dispensing data) [6, 17]. In addition, as this study is cross-sectional in design, no conclusions about an association between cause and effect can be taken. Even though the patients of this study were recruited from the main towns and the capital city where the majority of the Latvian population live, it seems that this could over-represent urban populations and underrepresent rural populations and the results cannot be generalized to the overall population of Latvian asthma patients.

Finally, the study addresses medication adherence related to frequency of medication use as per doctors’ prescription but does not address medication non adherence due to incorrect inhaler technique which is another important factor for receiving the appropriate dose of medicine.

## Conclusion

In conclusion, screening of asthma patients to assess their beliefs about their asthma medication may help to identify those ones to benefit from interventions targeting their concerns in order to improve treatment adherence. Many of the common beliefs of the Latvian asthma patients could be modified by educational interventions performed within the primary health-care system. This may have the potential to significantly reduce the economic burden related to poor asthma control and improve the quality of life of asthma patients in a cost-effective way.

## Data Availability

The datasets used and analyzed during the current study are available from the corresponding author on reasonable request.
